# Supplementary material for: Investigation of the Phase Transition Mechanism in LiFePO4 Cathode Using In Situ Raman Spectroscopy and 2D Correlation Spectroscopy during Initial Cycle
Source: Molecules. 2019 Jan 14;24(2):291. doi: 10.3390/molecules24020291 (PMC6359707; doi:10.3390/molecules24020291)
Supplement: Supplementary file 1 [file molecules-24-00291-s001.pdf]

Supporting information

Investigation of the phase transition mechanism in $LiFePO_4$ cathode using *in situ* Raman spectroscopy and 2D correlation spectroscopy during initial cycle

Yeonju Park[1], Soo Min Kim[1], Sila Jin[1], Sung Man Lee[2], Isao Noda[3,*], and Young Mee Jung[1,*]

[1] Department of Chemistry, Institute for Molecular Science and Fusion Technology, Kangwon National University, Chuncheon 24341, Korea; ymjung@kangwon.ac.kr

[2] Department of Nano Applied Engineering, Kangwon National University, Chuncheon 24341, Korea; smlee@kangwon.ac.kr

[3] Department of Materials Science and Engineering, University of Delaware, Newark, DE 19716, USA; noda@udel.edu

* Correspondence: noda@udel.edu (I. Noda), ymjung@kangwon.ac.kr; Tel.: +82-33-250-8495 (Y.M. Jung)

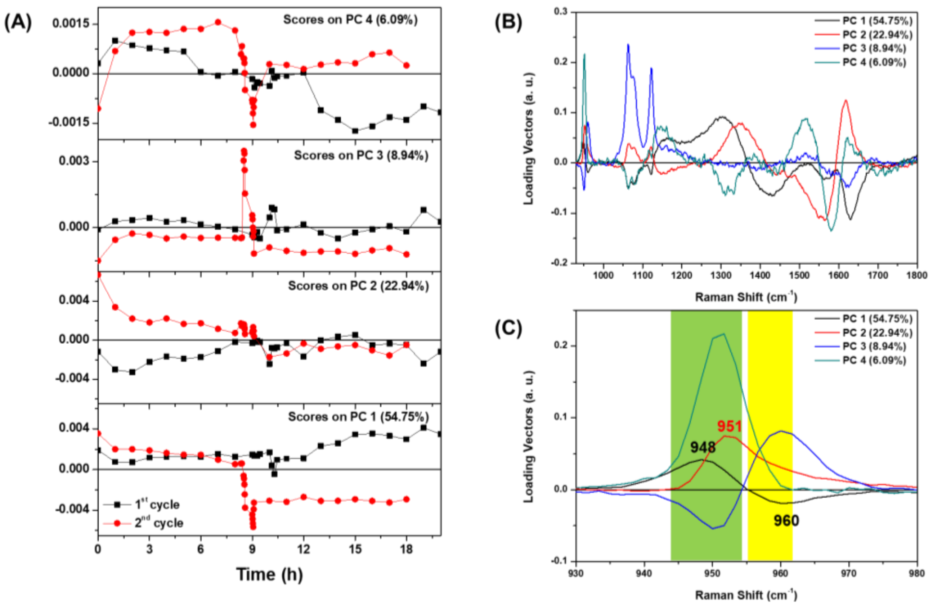

Figure S1. PAC results for *in situ* Raman spectra of $LiFePO_4$ cathode during the charging-discharging processes in the first and second cycles. The plots of the first four (A) scores, (B) loading vectors, and (C) loading vectors of 930-980 cm^{-1} region.

Table S1. Structural changes appeared voltage and time during the second charging-discharging processes

Index \ Li-ion cell No.	**No.207**	**No.215**	**No.222**	**No.225**	**No.228**	**No.239**	**No.242**
Voltage appeared spectral changes	3.65 V	3.66 V	3.69 V	3.59 V	3.69 V	3.61 V	3.63 V
Charging time	11 h 44 min	9 h 8 min	9 h	9 h 7 min	8 h 52 min	8 h 53 min	8 h 56 min
Time appeared spectral changes	11 h 33 min	8 h 5 min	8 h 48 min	8 h 50 min	8 h 38 min	8 h 30 min	8 h 42 min
Measurement interval at near the end of charging /at all charging-discharging processes	2 min /1 h	2 min /1 h	2 min /1 h	2 min /1 h	2 min /1 h	2 min /1 h	2 min /1 h
One cycle time	23 h 18 min	18 h	17 h 41 min	17 h 43 min	17 h 26 min	17 h 33 min	17 h 26 min

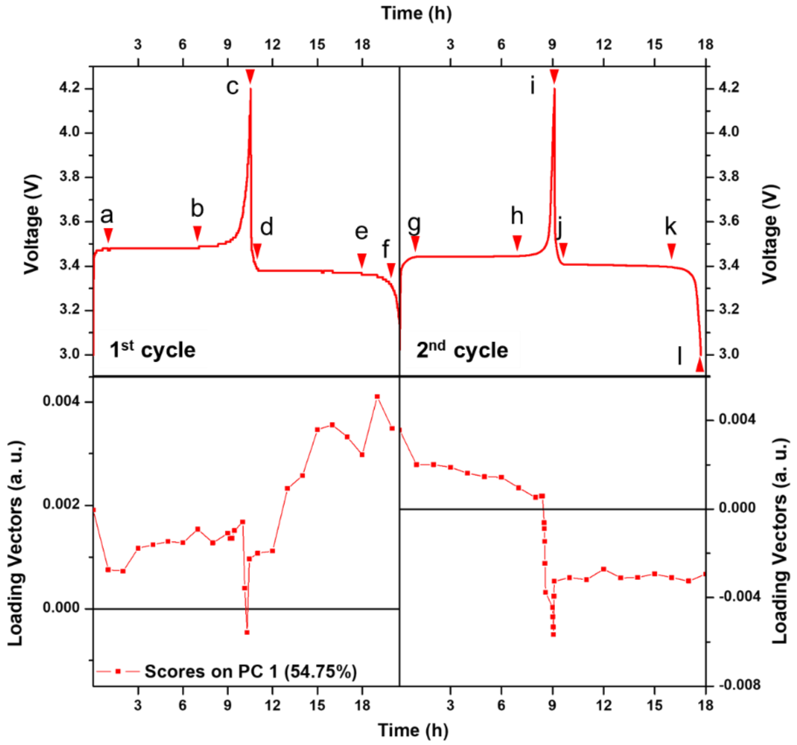

Figure S2. (A) Charging and discharging profiles during the first and second cycles of a $LiFePO_4$/Li cell; a-f: the first cycle (a: 1 h, b: 7 h ,c: 10 h 30 min (end of charge), d: 11 h, e: 18 h ,f: 20 h (end of discharge)) and g-i: the second cycle (g: 1 h, h: 7 h , i: 9 h 8 min (end of charge), j: 10 h, k: 15 h ,l: 18 h (end of discharge)). (B) Score plot of PC 1.

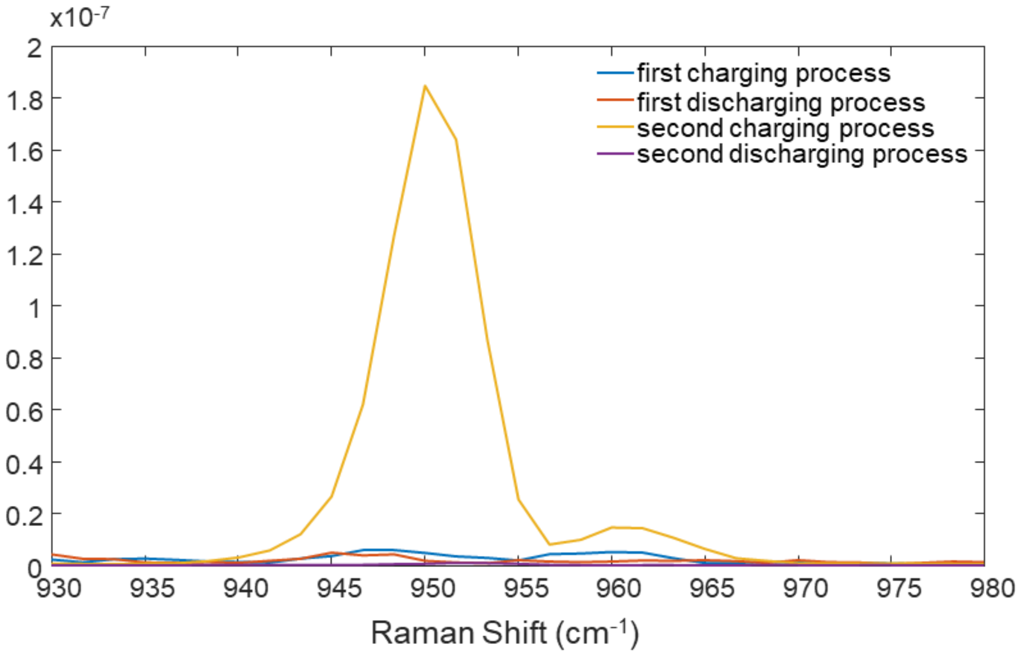

Figure S3. Power spectra extracted along the diagonal line on the synchronous 2D correlation spectra as shown in Figures 3(A), 3(C), 4(A), and 4(C).
